# Face Avulsion and Degloving

**Published:** 2014-01

**Authors:** Nikhil Panse, Parag Sahasrabudhe, Namrata Joshi

**Affiliations:** Department of Plastic Surgery, BJ Medical College and Sassoon Hospital, Pune, India

**Keywords:** Face, Degloving, Eyelid, Nose, Avulsion

## Abstract

There have been sparse reports in literature of avulsion and degloving injuries of individual areas of face like the nose, eyelids, ear and even mandible. Hemi-facial degloving is extremely rare. We present a case of post-assault degloving of the nose, part of forehead with anterior wall of frontal sinus, entire upper and lower eyelids and the cheek. Proper planning and staging of the surgical procedures and use of local flaps, meticulous and proper alignment of tissues gave us good aesthetic and functional outcome with a satisfied patient.

## INTRODUCTION

Facial reconstruction has always been a challenge to the reconstructive surgeon. With multiple areas of degloving, the reconstruction became even more challenging.^1^ This study post-traumatic degloving of multiple areas of the face. Goals of reconstruction included restoration of a functional and aesthetic nasal and ocular unit; restoration of an aesthetic eyebrow and forehead unit with minimal morbidity to the patient. 

## CASE REPORT

**Fig. 1:**
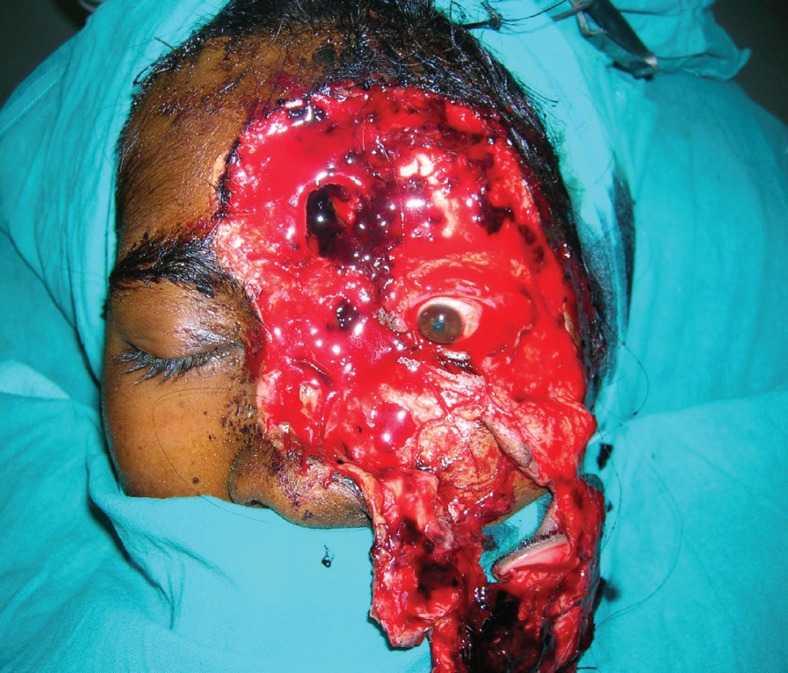
Hemifacial degloving in the patient

A 25 years old female presented with alleged history of assault by husband over the left hemi-face by a sickle two days after the trauma. There was degloving and avulsion of the left upper hemi-face including the nose, forehead skin and eyebrow on the left side, upper and lower eyelids and part of the cheek. The globe as well as eyesight was intact. The extra ocular movements were present (Figure 1).

A detailed evaluation under anesthesia revealed that there was loss of the anterior cortex of the frontal sinus, and loss of the nasal bones as well. The part of the frontal sinus was attached to the degloved skin flap. Part of frontal bone on left side was exposed. The wound was contaminated with small amount of mud particles.

A thorough debridement was undertaken, and the devitalized tissue was excised. An attempt was made to reposition the degloved flap and the structures to their respective positions. It was like solving a jigsaw puzzle. Meticulous suturing was done to relocate the avulsed structures to their respective positions. Medial and lateral canthopexies were done to relocate the eyelids at their respective positions under appropriate tension. 

**Fig. 2:**
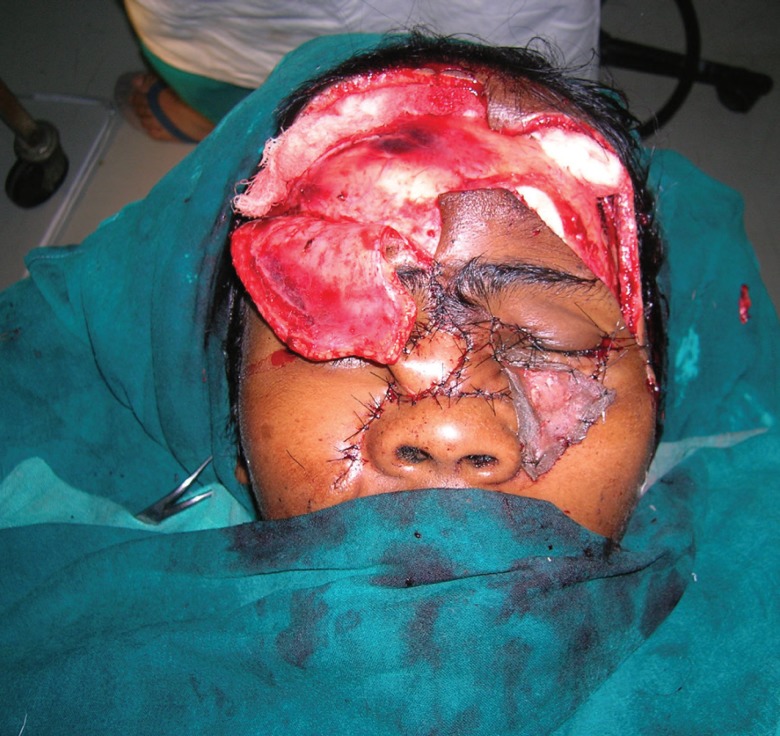
Intra-operative picture showing canthopexy, harvested nasolabial flap and forehead flap

**Fig. 3:**
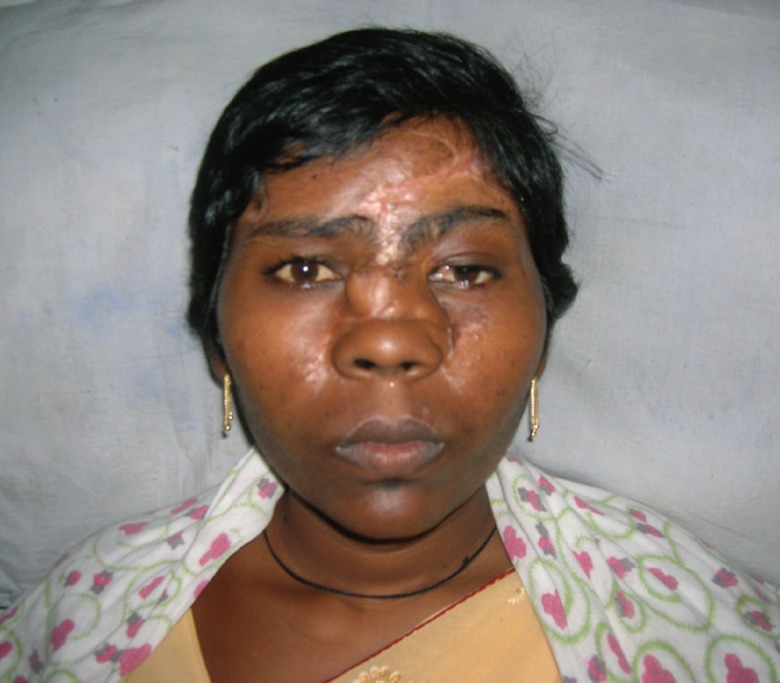
Well settled flaps and position of medial and lateral canthi

There was significant loss of tissue in the left forehead region, over the nasal dorsum and cheek areas. The exposed frontal sinus was curetted thoroughly to scrape all the mucosa and the cavity was plugged with pieces of the outer cortex of the frontal sinus. A forehead flap was raised based on the right supraorbital and supratrochlear vessels and transposed medially to cover the exposed frontal sinus and exposed frontal bone. A proximally based nasolabial flap was used to cover the skin defect on the dorsum and right lateral wall of the nose. A small defect on the cheek region was split skin grafted. The post operative course was uneventful (Figure 2 and 3).

**Fig. 4:**
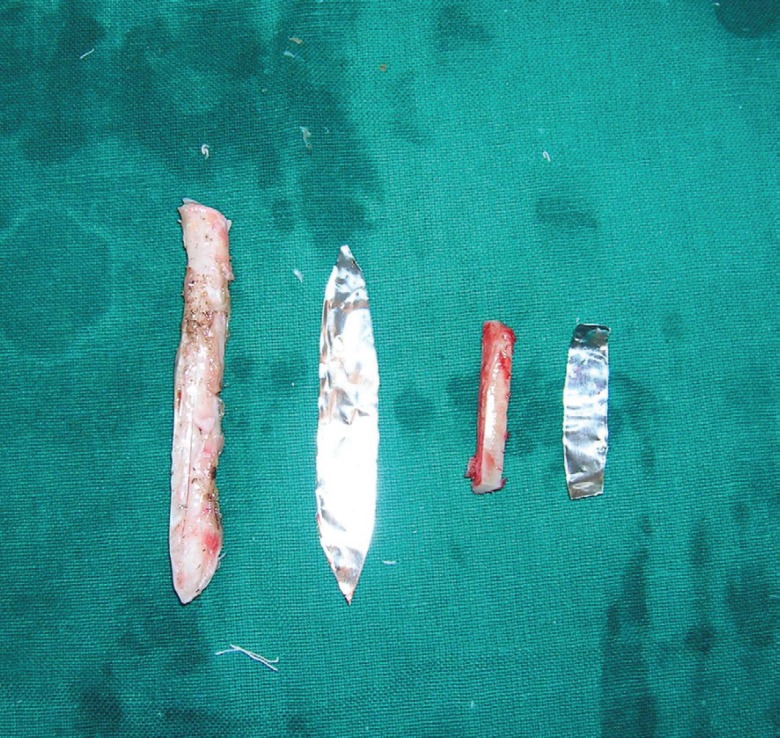
Harvested costal cartilage for nasal dorsum and columella augmentation

After four weeks, costal cartilage graft was used for nasal augmentation (Figure 4). Costal cartilage was placed in an L shaped manner for augmentation of the columella and dorsum of the nose. The placement was done through a small stab incision over the columella. The fixation of the grafts was done as by the technique described by us previously.^1^ Thinning of the Nasolabial flap was done in the same setting. The post operative course was uneventful.

**Fig. 5:**
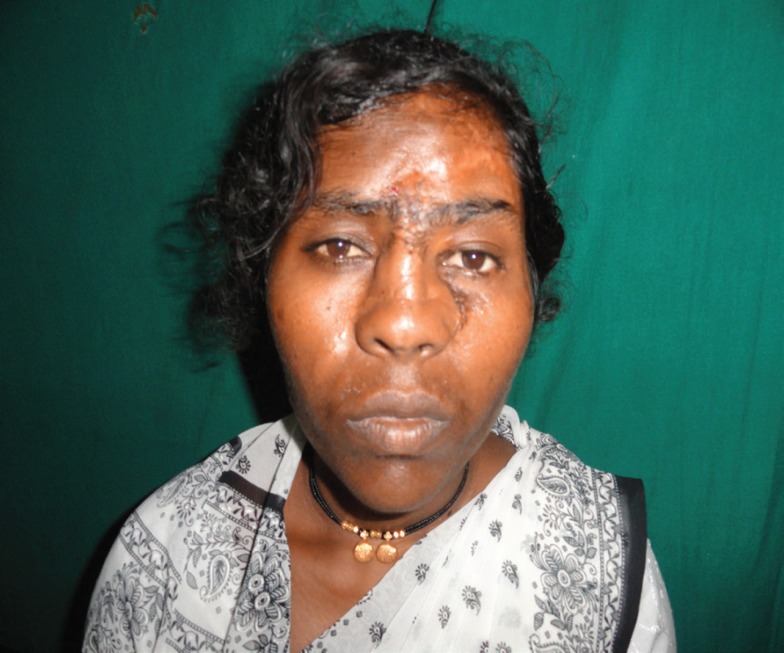
After nasal dorsum augmentation and debulking of nasolabial flap

One year after the trauma, and after two surgeries, we had a satisfied patient with no functional deficit (Figure 5). There were no nasal or ocular complaints. Eye opening and closure were normal. We felt that the cosmetic outcome was suboptimal, and offered the patient the options of scar revision over the forehead, staged removal of skin graft over the cheek, and a more appropriate eyebrow positioning. However the patient was satisfied and was not desirous of any other surgical procedures suggested to her.

## DISCUSSION

The incidence of craniomaxillofacial injuries as a result of interpersonal violence is increasing in the general population. However, the incidence of female assault cases is increasing disproportionately and women constitute 20 to 25% of facial-trauma victims.^2^ Domestic violence represents a leading cause of trauma among females. Recent research has suggested that violence against females often has a context, mechanism, and perhaps even injury type distinct from those of male cases. 

Assault trauma in females is a major public health problem. Although, we do not have exact statistics for the developing world, in the US domestic violence in females cost the health care system 3 to 5 billion Dollars per year, and devastate millions of lives.^3^ Most domestic attacks occur without the use of weapons, and perhaps, the attacker uses more restraint than that of the perpetrator in the violent acts predominating among males (e.g., gang related assaults, altercations).^4^^-^^6^ However, our patient allegedly suffered a sharp assault by a sickle at the hands of her husband who was in a drunk state.

There have been sparse reports in literature of avulsion and degloving injuries of individual areas like the nose,^7^ eyelids,^8^ and even mandible.^9^ But an extensive degloving avulsion injury of the magnitude encountered by us is extremely rare. To the best of our knowledge, this is the first reported case of a degloving avulsion injury of hemi face.

Hallock *et al.*^7^ emphasized that degloving injuries of the external nose are severe soft tissue avulsions requiring meticulous repair to prevent airway embarrassment and to provide the best aesthetic result. Usually the underlying nasal scaffolding remains intact, but associated facial fractures or other facial injuries are common and must not be overlooked.

Bergman *et al.*^8^ described an upper face and orbit degloving injury wherein a 70-year-old woman’s 2 pet dachshunds chewed her upper face and bilateral periorbital areas. A subtotal exenteration was performed on her left orbit and a temporoparietal fasciocutaneous flap was used to reconstruct her right orbit with buccal mucosa replacing both the bulbar and palpebral conjunctiva.

Oluremi *et al.*^10^ described the challenges faced in case of multiple midface degloving injury with multiple fractures and airway obstruction. They emphasized on the importance of airway management, interdisciplinary care and nutrition for optimal results in such patients. Isolated eyelid avulsion injuries are itself very rare^11^ that can be caused by motor vehicle accidents, dog bites, or human bites.^12^ Eyelid avulsions are repaired by suturing after a CT scan is performed to determine where damage to the muscles, nerves, and blood vessels of the eyelid has occurred.^12^ In our patient, the extra-ocular movements were normal, and so we proceeded without undertaking a computed tomography imaging.

Although on primary examination, the wound seemed ghastly and difficult, once we started to place the parts to their respective positions, we realized that the defects created were amenable to local flaps. Local tissues are always the best option for resurfacing the face.^13^ We used the available forehead, and nasolabial flaps for resurfacing the defects. Soft tissue reposition was done in layers because improper repositioning of soft tissues predispose the site to deformities with subsequent adverse effects on the aesthetics of the final outcome.^14^

The challenge in treating patients with extensive craniofacial trauma is to achieve pre-injury facial profile and most importantly facial projection. Also traumatic lesions of the soft tissue of the face have important social implications, which is why these injuries must be managed aggressively. Wound contamination was a very big risk in spite of thorough debridement, appropriate antibiotics and post operative care. It was because of this risk that we postponed the nasal augmentation by costal cartilage to the next stage. An L shaped strut gave us an acceptable facial projection.

In our case, we did not encounter any airway obstruction, but avulsion injuries to mid-face are always at a very high risk of airway obstruction which must be addressed on an urgent basis as and when necessary. This nature of multiple degloving injuries make this case unique and worth sharing.

Facial degloving injury is a complex reconstructive challenge which may require a staged approach for optimal cosmetic and functional outcome. Appropriate airway maintenance where necessary, radicle debridement, meticulous, layered and proper alignment of tissues to their respective positions are instrumental in achieving good results. Proper planning and staging of the surgical procedures and use of local flaps gave us good aesthetic and functional outcome with a satisfied patient.

## Footenot

Please cite this paper as:

Panse N, Sahasrabudhe P, Joshi N. Face Avulsion and Degloving. World J Plast Surg 2014;3(1):64-67.
